# A Horned Viper Bite Victim with PRES

**DOI:** 10.1155/2017/1835796

**Published:** 2017-04-10

**Authors:** Ahmed Mustafa Ibrahim, Tarek Talaat ElSefi, Maha Ghanem, Akram Muhammad Fayed, Nesreen Adel Shaban

**Affiliations:** Faculty of Medicine, Alexandria University, Alexandria, Egypt

## Abstract

Neurological complications of snake bites have been well documented in the literature as neuromuscular paralysis and cerebrovascular complications; posterior reversible encephalopathy syndrome was rarely described. A 23-year-old lady presented near full term of her pregnancy with a horned snake* Cerastes cerastes* bite; after successful delivery she started complaining of altered mental status and visual disturbance with ulceration over the site of the snake bite. On admission, the patient had Glasgow Coma Score of 12, blood pressure 130/80 mmHg, temperature 38°C, sinus tachycardia at 120 beats per minute, severe dehydration, and reduction in visual acuity to “hand motion” in both eyes with poor light projection and sluggish pupillary reactions. CT brain was not conclusive; MRI revealed features of PRES. Treatment was mostly supportive within one week; the patient regained consciousness; visual disturbance, however, persisted. This patient as well as the few previously described cases highlights PRES as a possible complication of snake bites.

## 1. Background

The sand horned vipers (genus* Cerastes*) are the best identified and most abundant venomous snakes of the deserts of North Africa and the Middle East [1]. Various sequelae of human envenomation have been documented, with coagulopathy, hemolysis, and disseminated intravascular coagulopathy being the commonest findings [1, 2]. Both ischemic and hemorrhagic strokes, as well as acute renal failure and pancreatitis, have also been described [1, 3–7]. Many toxins have been identified from* Cerastes cerastes* venom. These include serine proteases and other thrombin-like enzymes (fibrinogenases) (IVa, Cerastocytin, Cerastotin, RP3 4, Afaâcytin, and Cerastase F-4), activators of platelet aggregation/agglutination (Cerastocytin, Cerastotin), inhibitors of platelet aggregation (IVa, Cerastatin, Cerastin), Factor X activators (calcium-dependent and calcium-independent serine proteases, Afaâcytin), a hemorrhagic protease (Cerastase F-4), and *α*/*β* fibrinogenase, which releases serotonin from platelets (Afaâcytin), and a phosphodiesterase exonuclease and a weakly toxic phospholipase A_2_, which could contribute to local tissue damage. Obviously some of these toxins have opposite actions which suggest that the clinical picture of the snake bite would depend on the exact composition of the venom of the particular snake with large intraspecies variations [1].

Neurological complications of snake bites, primarily neuromuscular paralysis, hypopituitarism, and strokes, have also been recorded in the literature [8–10].

While intracranial hemorrhage in snake bite patients has been clearly attributed to the coexisting coagulopathy, the process by which ischemic strokes occur is still a matter of debate, with various mechanisms currently proposed such as endothelial injury, hypercoagulability, autoimmune vasculitis, thrombotic microangiopathy, and systemic hypotension [8, 9].

Posterior reversible encephalopathy syndrome (PRES) is a neurological disease whose pathophysiology is still not fully understood. Various theories have been put forward in an attempt to explain the mechanism by which PRES occurs, including endothelial injury and hypoperfusion [11, 12]. Only a handful of case reports have described the occurrence of PRES with snake bites as well as other animals' [13].

In this case report we describe the case of a young lady who presented to us with neurological symptoms following a horned sand viper bite that was later diagnosed as PRES.

## 2. Case Report

A previously healthy 23-year-old lady in her 37th week of pregnancy (G1P0A0) was bitten in her left leg by a snake that was later identified as the horned viper* Cerastes cerastes*. She developed pain and blisters at the site of the bite. A polyvalent antivenin was administered to the patient and she was transferred to a hospital in Tobruk, Libya. She presented to the hospital fully conscious. Her vital signs showed a blood pressure and heart rate of 110/70 mmHg and 130 beats per minute, respectively. Investigations were as follows: hemoglobin (Hb) 9.2 gm/dl, White Blood Cell (WBC) count 9.3 × 10^3^, platelets (plt) 105 × 10^3^, and INR 2.7. Fearing for fetal adverse events [14] and given that she was already at full term, pregnancy was terminated by caesarean section after fresh frozen plasma transfusion.

Over the following week, the patient started to become feverish and obtunded. Gradual loss of vision ensued. No seizures, headache, or paresis occurred. A brain CT scan was done but was unremarkable. Edema of the left leg worsened, with the ulcer at the bite site later on turning gangrenous. The patient had stable blood pressure throughout her stay in Libya with no evidence of preeclampsia. Urine analysis tested negative for proteinuria. The preliminary diagnosis was septic encephalopathy and the patient was transferred to our center, El-Ekbal Hospital, a private tertiary care facility in Alexandria, Egypt.

On admission, the patient was stuporous with a Glasgow Coma Score of 12 (E3M5V4). Vital signs were as follows: blood pressure 130/80 mmHg, temperature 38°C, respiratory rate 15 breaths per minute, and sinus tachycardia at 120 beats per minute which persisted even after adequate fever control and correction of anemia.

Neurological examination revealed a reduction in visual acuity to “hand motion” in both eyes with poor light projection and sluggish pupillary reactions.

On assessing the volume status, the patient was found to be severely dehydrated with an initial CVP reading of −3 and −2 cm H_2_O. The C-section wound did not show any signs of infection; however her left leg displayed a gangrenous ulcer.

Her initial investigations were as follows: Hb 8.3 g/dL, WBC 8.1 × 10^3^, Plt 63 × 10^3^/uL, INR 1.1, Na^+^ 142.6 mmol/L, K 3.21 mmol/L, Mg^+2^ 0.9 mg/dL, total bilirubin 0.7 mg/dL, creatinine 0.76 mg/dL, and urea 35.3 mg/dL. Urine analysis revealed a specific gravity of 1005, albumin: nil, urobilinogen: normal trace, pH 6.5, pus cells 2-3/HPF, RBCs 8–10/HPF, SGOT 11 U/L, SGPT 35 U/L, and total bilirubin 0.7 mg/dl.

A hypercoagulability panel was ordered. The patient tested positive for Factor V Leiden gene mutation and methylenetetrahydrofolate reductase (MTHFR) gene mutation and negative for protein C, protein S, antinuclear antibodies, and antiphospholipid antibodies.

Differential diagnosis of the altered sensorium was either a neurological complication of the snake bite or sepsis associated encephalopathy. Quantitative C-reactive protein and procalcitonin levels were 13.7 mg/L and 0.2 ng/mL, respectively, which correlated with mild-to-moderate localized bacterial infection rather than sepsis [19].

**Figure 1:**
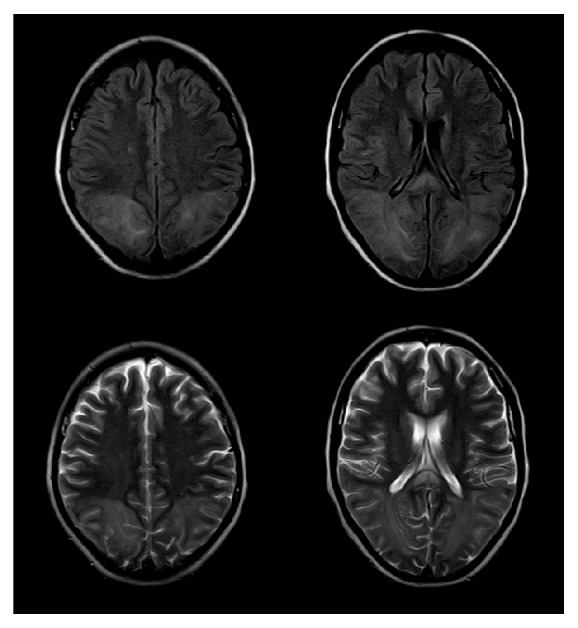
Bilateral symmetrical bright T2 and FLAIR white matter intensity involving the posterior parietal sections of both cerebral hemispheres, as well as the splenial segment of the corpus callosum.

**Figure 2:**
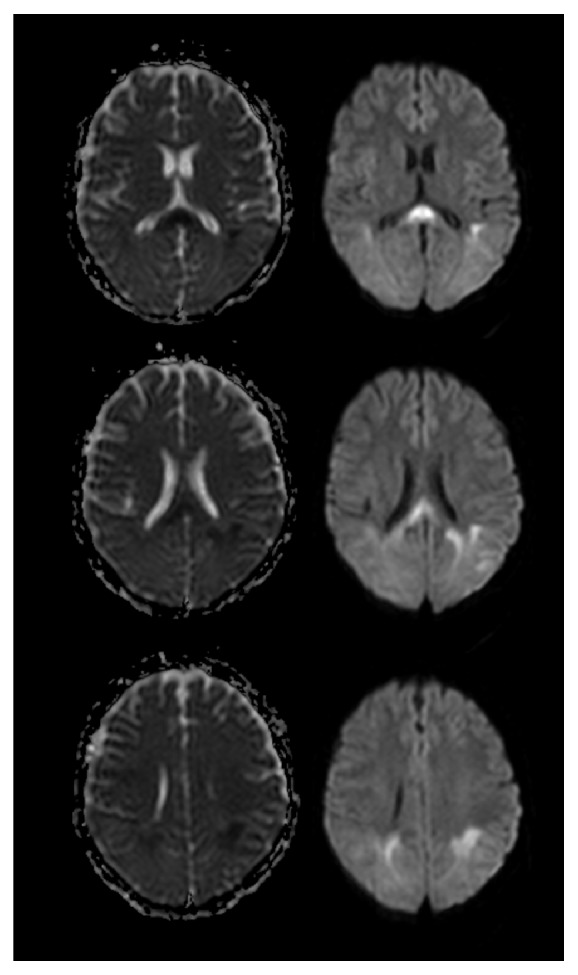
DW/ADC map series showing patchy diffusion restriction in posterior parietal sections as well as splenial segment of corpus callosum.

A follow-up brain CT illustrated a very faint hypodense area in the posterior part of the parietal and occipital lobes. MRI revealed a bilateral symmetrical bright T2 and FLAIR white matter intensity involving the posterior parietal sections of both cerebral hemispheres, as well as the genu and splenial segments of the corpus callosum, with patchy diffusion restriction in the DW/ADC map series: features of PRES either with atypical findings of patchy diffusion restriction or complicated with ischemia. Magnetic resonance venography (MRV) was normal (Figures 1 and 2).

Doppler US of the left lower limb arterial system revealed a monophasic waveform in the posterior tibial and the dorsalis pedis arteries with good pulsatility and adequate peak systolic velocity, along with a hyperdynamic inflammatory pattern. Venous Doppler US showed superficial thrombophlebitis.

Treatment was mostly supportive: fluid and electrolyte replacement, antibiotics and debridement of the necrotic tissue, clopidogrel, folate, and vitamin B complex for MTHFR deficiency. Diltiazem was prescribed for inappropriate tachycardia and risperidone for agitation and blood transfusion for anemia.

Within one week of hospital stay the patient remained normotensive, her hyperthermia was rapidly controlled as well as the sinus tachycardia, and the patient regained consciousness and hemoglobin and platelet levels normalized. Blindness, however, persisted. She was discharged to home in Libya, no follow-up MRI was done, and the patient reported three months later still having visual impairment though her vision is improving.

## 3. Discussion

Snake venom contains a mixture of cytotoxic, hypotensive, neurotoxic, and anticoagulant substances and varies in composition according to the species of the snake [8].

Neurotoxins are a major component of many venoms. These toxins cause paralysis by affecting the neuromuscular junction at presynaptic or postsynaptic levels. Presynaptic neurotoxins inhibit the release of acetylcholine from the presynaptic neuron. Postsynaptic neurotoxins are three-finger protein complexes, which have a curare-like action, causing a reversible blockage of acetylcholine receptors. Some venoms contain both types of neurotoxins, producing complex blockages of neuromuscular transmission [8]. This blockade presents as acute muscle weakness or, more dangerously, respiratory muscle failure which is potentially fatal [9].

Major neurological complications were described in cases of snake bites, most notably ischemic and hemorrhagic infarctions. These are most likely related to the components of the toxins that interfere with blood coagulation and cause bleeding or clotting [8]. While intracranial hemorrhages are related to pathology of hemostatic factors as decreased platelets or a severe consumptive coagulopathy, the mechanism underlying ischemia is still not established. Proposed mechanisms include hypercoagulability, endothelial damage, vasculitis, and hypotension. The presence of multiple cerebral infarctions in most of the cases supports the hypothesis that infarctions are most likely related to the prothrombotic effects of the venom as well as endothelial damage [8], with the latter being the proposed mechanism by which PRES occurs in snake bite victims.

PRES is characterized by a variety of neurological symptoms including encephalopathy (50–80%), seizures (60–75%), headache (50%), visual disturbances (33%), focal neurological deficits (10–15%), and status epilepticus (5–15%) that is accompanied by a potentially reversible imaging pattern of subcortical vasogenic brain edema [11].

Two hypotheses were suggested to explain the pathogenesis of PRES: (1) hypertension exceeding the limits of autoregulation, causing breakthrough brain edema, and (2) hypertension leading to cerebral autoregulatory vasoconstriction, ischemia, and consequent brain edema [12].

Moderate-to-severe hypertension is encountered in about 70% of patients with PRES at presentation, and emergent hypertension treatment is associated with symptom improvement in many cases. Nevertheless, a significant number of PRES patients presented without hypertension, including the case we are discussing in this report. Data supporting hyperperfusion are scarce and the extent of brain edema does not correlate with the severity of hypertension [12]. The aforementioned findings delineate only two of the several problems affecting this theory.

Another theory proposed to explain PRES is the role of endothelial dysfunction. In the majority of patients who develop PRES; a complex underlying systemic inflammatory process is present as T-cell activation, inflammatory cytokine production, endothelial surface antigens activation, endothelial antibodies, immune system antigens, and VEGF elevation. Cytokines (TNF-a IL-1) upregulate endothelial surface antigens (P-selectin, E-selectin, ICAM-1, and VCAM-1) and increase leukocyte adherence (trafficking) leading to microvascular dysfunction. Enhanced systemic endothelial activation, leukocyte trafficking, and vasoconstriction, alone or in combination, will result in brain and systemic hypoperfusion [20].

Evidence of vasculopathy and cerebral hypoperfusion is present on current imaging studies and is likely reflected in the watershed appearance of the vasogenic edema that develops in PRES, which may support the theory of systemic inflammatory response leading to hypoperfusion [12, 20]. Other minor hypotheses put magnesium as part of the pathogenesis of PRES, either as a cause or as an aggravating factor. Noteworthy is that our patient had persistent hypomagnesemia [21].

PRES is associated with conditions as severe hypertension, blood pressure fluctuations, renal failure, eclampsia, preeclampsia, autoimmune disorders, and cytotoxic drugs. Few accounts described the occurrence of PRES in victims of animal bites [13] as scorpions [22], wasps [23], and snakes.

Four cases in the literature described the occurrence of PRES with snake bites (Table 1). The first case, described by Delgado and Del Brutto, was that of an 18-year-old man bitten by* Bothrops asper*. Two days after receiving the antivenin, he developed disturbance in the level of consciousness, respiratory distress, and coagulopathy; a brain CT with IV contrast revealed bilateral hypodensities that resolved upon resolution of the symptoms [15].

Chaudhary et al. illustrated the second case, being that of a 40-year-old woman bitten by an unknown species of snake. Unconsciousness and coagulopathy developed 30 minutes after the bite; MRI revealed signal intensity alteration in the caudate and lenticular nuclei as well as the thalami, along with involvement of the cortical rim suggestive of an asymmetrical leucoencephalopathy with the DW image excluding ischemia. Improvement of the general condition followed administration of the antivenin. Nonetheless, a parkinsonism-like state ensued which required long term treatment with a carbidopa-levodopa combination [16].

The third case was published in a letter by Varalaxmi et al. Following a bite by a pit viper, a 45-year-old man developed renal impairment. On the second day, the patient developed cortical blindness and headache. No antivenin was given. Brain MRI revealed cortical bilateral hyperintensity affecting the parietal, occipital, and subcortical white matter and a diagnosis of PRES was established. The neurological symptoms started to improve within 12 hours with complete resolution occurring 24 hours after the bite [17].

The fourth case, a 10-year-old boy bitten by an Indian Krait, was reported by Kaushik et al. The patient presented with respiratory distress, disturbed level of consciousness, sluggish light reflexes, uncontrolled hypertension, and autonomic disturbances. Improvement of the symptoms occurred 3 days after receiving the antivenin and the patient was discharged. Six days later, he presented with focal status epilepticus, uncontrolled hypertension, and visual disturbance. Brain MRI revealed hyperintensity on T2-FLAIR images in the parietooccipital subcortical white matter with a picture of PRES. His vision returned to normal within 2 days, and he was discharged on antiepileptic and antihypertensive medications. On follow-up, his blood pressure normalized and his antihypertensive medications were stopped. Repeated MRI studies revealed resolution of the findings [18].

What stands out in these cases as well as ours is that there was no hypertension in 4 of the 5 patients except Kaushik et al.'s [18]. Two of the cases had other clinical conditions that may explain the occurrence of PRES; the patient of Kaushik et al. [18] had uncontrolled hypertension and the patient of Varalaxmi et al. had renal impairment. Both conditions are known to be associated with PRES [11]. Our patient's symptoms developed in the early postpartum period. While eclampsia and preeclampsia are the most common conditions leading to PRES [24], our patient had neither hypertension nor proteinuria, which excludes preeclampsia as a cause of the disorder.

Two out of the five patients received antivenin prior to the development of the neurological symptoms. If the initial symptoms of the patient of Kaushik et al. [18] were not part of PRES, that would make them 3. Antivenin is known to have possible adverse neurological effects [8]. Four of the patients at one point or another received antivenin.

Although the exact mechanism of PRES is not clear in these cases, the temporal relation with the snake bites makes the association between the two very probable.

One of the remarkable features of the case we present here is the restricted diffusion pattern revealed in the MRI. Restricted diffusion is one of the atypical presentations of PRES. In a study by McKinney et al. it was the second most common atypical presentation of PRES [25]. It indicates the presence of cytotoxic edema rather than the vasogenic edema associated with PRES [26]. This finding was traditionally attributed to the development of ischemia on top of PRES and was regarded as a poor prognostic factor due to the irreversibility of the condition [26–28]. Other case reports and series suggest the contrary since reversible restricted diffusion was noted in cases of subarachnoid hemorrhage and transient ischemic attacks. Recent data suggests that the same may be true for PRES with patches of restricted diffusion [29–31]. In a study by Wagih et al., neither the extent of the lesion nor any imaging variable, such as the apparent diffusion coefficient (ADC), had statistically significant correlation with the reversibility of the disease [31], and other multicentric studies as the study done by Pande et al. also found that DWI, even with ADC maps, had limitations in predicting the course of PRES [32].

Despite resolution of most of our patient's symptoms, the persistence of vision loss for months after the bite may favor the possibility of irreversibility.

The relation between the patient's hypercoagulable state and PRES or the restricted diffusion pattern is unclear; scanty data are found in literature regarding the association between PRES and hypercoagulability [33], hypercoagulability may have been an added factor of endothelial injury in the patient.

## Figures and Tables

**Table 1:** A summary of the clinical presentation of five cases of PRES in patients with snake bites.

	Type of snake	Onset	Antivenin Received	Manifestations								Reversibility
				Coagulopathy	Renal impairment	Respiratory failure	Hypertension	Neurological symptoms				
								Seizures	Visual disturbance	DLC	Motor disorders	
Our patient	*Cerastescerastes*	Within a week	Yes, before symptoms	Yes	No	No	No	No	Yes	Yes	No	Partially
Delgado and Del Brutto [15]	*Bothropsasper*	2 days	Yes, before symptoms	Yes	No	Yes	No	Yes	No	Yes	No	Yes
Chaudhary et al. [16]	Not identified	30 min	Yes, after symptoms	Yes	No	No	No	No	No	Yes	Yes	Partially
Varalaxmi et al. [17]	Pit viper	2 days	No	No	Yes	No	No	No	Yes	Yes	No	Yes
Kaushik et al. [18]	*Bungaruscaeruleus*	hours	Yes, after symptoms	Not clear	Not clear	Yes	Yes	Yes	Yes	Yes	Yes	Yes

## Additional Points

*Key Learning Points.* (1) Due to the complexity of the structure of snake venom, all possible presentations must be anticipated no matter how rare they are. (2) The association between PRES and venomous animal bites may cast some light on the pathogenesis of the condition and thus requires further studying. (3) Most snake bite victims are inhabitants of remote and rural areas. A competent system for providing the essential primary care and referral must be established.
